# Nuclear and mitochondrial population genetics of the Australasian arbovirus vector *Culex annulirostris* (Skuse) reveals strong geographic structure and cryptic species

**DOI:** 10.1186/s13071-024-06551-8

**Published:** 2024-12-04

**Authors:** William Atherton, Luke Ambrose, James Wisdom, Bryan D. Lessard, Nina Kurucz, Cameron E. Webb, Nigel W. Beebe

**Affiliations:** 1 School of the Environment, University of Queensland, Brisbane, Australia; 2 Department of Climate Change, Energy, the Environment and Water, Australian Biological Resources Study, Canberra, Australia; 3 CSIRO Australian National Insect Collection, Canberra, ACT Australia; 4 Medical Entomology, Centre for Disease Control, Public Health, NT Health, Darwin, NT Australia; 5 Department of Medical Entomology, NSW Health Pathology, Sydney, Westmead Hospital, Westmead, NSW Australia; 6 School of Medical Sciences, Faculty of Medicine and Health, University of Sydney, Camperdown, NSW Australia

## Abstract

**Background:**

The mosquito *Culex annulirostris* Skuse (Diptera: Culicidae) is an important arbovirus vector in Australasia. It is part of the *Culex sitiens* subgroup that also includes *Cx. palpalis* and *Cx. sitiens*. Single locus mitochondrial and nuclear DNA sequencing studies suggest that *Cx. annulirostris* consists of a complex of at least two species. We tested this hypothesis by analysing both nuclear microsatellite data and additional mitochondrial sequence data to describe the population genetics of *Cx. annulirostris* through Australia, Papua New Guinea (PNG) and the Solomon Archipelago.

**Methods:**

Twelve novel microsatellite markers for *Cx. annulirostris* were developed and used on over 500 individuals identified as *Cx. annulirostris* by molecular diagnostics. Ten of the 12 microsatellites then used for analysis using Discriminant Analysis of Principal Components, a Bayesian clustering software, STRUCTURE, along with estimates of Jost’s D statistic that is similar to *F*_*ST*_ but better suited to microsatellite data. Mitochondrial cytochrome oxidase I (COI) DNA sequence were also generated complementing previous work and analysed for sequence diversity (Haplotype diversity, Hd and Pi, π), Tadjima’s D, and pairwise *F*_*ST*_ between populations. An allele specific molecular diagnostic with an internal control was developed.

**Results:**

We confirm the existence of multiple genetically and geographically restricted populations. Within mainland Australia, our findings show that *Cx. annulirostris* consists of two genetically and geographically distinct populations. One population extends through northern Australia and into the south-east coast of Queensland and New South Wales (NSW). The second Australian population occurs through inland NSW, Victoria, South Australia, extending west to southern Western Australia. These two Australian populations show evidence of possible admixture in central Australia and far north Queensland. Australia’s Great Dividing Range that runs down southeast Australia presents a strong gene-flow barrier between these two populations which may be driven by climate, elevation or river basins. In PNG we find evidence of reproductive isolation between sympatric cryptic species occurring through PNG and Australia’s northern Cape York Peninsula. A PCR-based molecular diagnostic was developed to distinguish these two cryptic species.

**Conclusion:**

This study adds to the growing body of work suggesting that the taxon presently known as *Cx. annulirostris* now appears to consist of at least two cryptic species that co-occur in northern Australia and New Guinea and can be distinguished by a ITS1 PCR diagnostic. The Solomon Islands population may also represent a distinct species but in light its geographic isolation and lack of sympatry with other species would require further study. Additionally, the mitochondrial and nuclear DNA evidence of population structure between geographic regions within Australia appears latitudinal and elevational driven and may suggest an additional subspecies in that hybridise where they overlap.

**Graphical Abstract:**

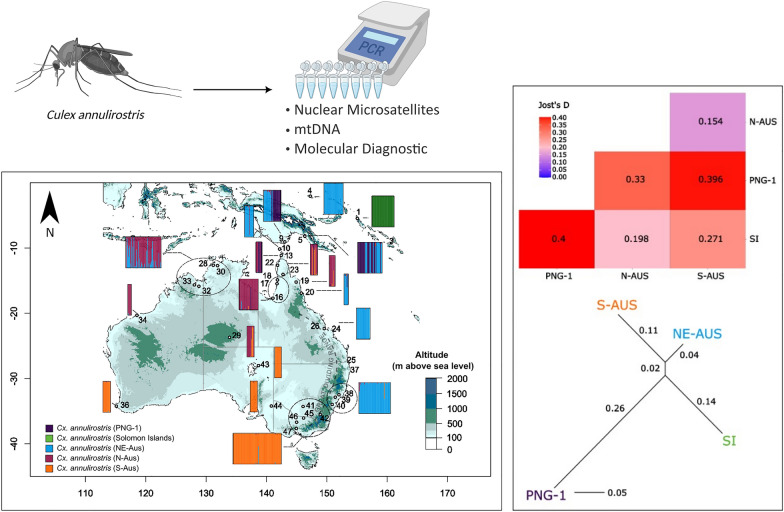

**Supplementary Information:**

The online version contains supplementary material available at 10.1186/s13071-024-06551-8.

## Background

Despite mosquitoes being vectors of pathogens that cause many potentially fatal and debilitating human and animal diseases, the diversity of these potentially highly speciose groups remain incomplete. The freshwater common banded mosquito *Culex annulirostris* Skuse (Diptera: Culicidae) occurs through the Australasian and Indo-Pacific region and transmits several endemic arboviruses through Australasia. These include the alphaviruses, Ross River and Barmah Forrest virus, as well as flaviviruses such as Kunjin-West Nile virus (KUNV), Japanese encephalitis virus (JEV) and Murray Valley encephalitis virus (MVEV [30, 40], the latter two which have had major recent outbreaks on mainland Australia [10, 35].

**Fig. 1:**
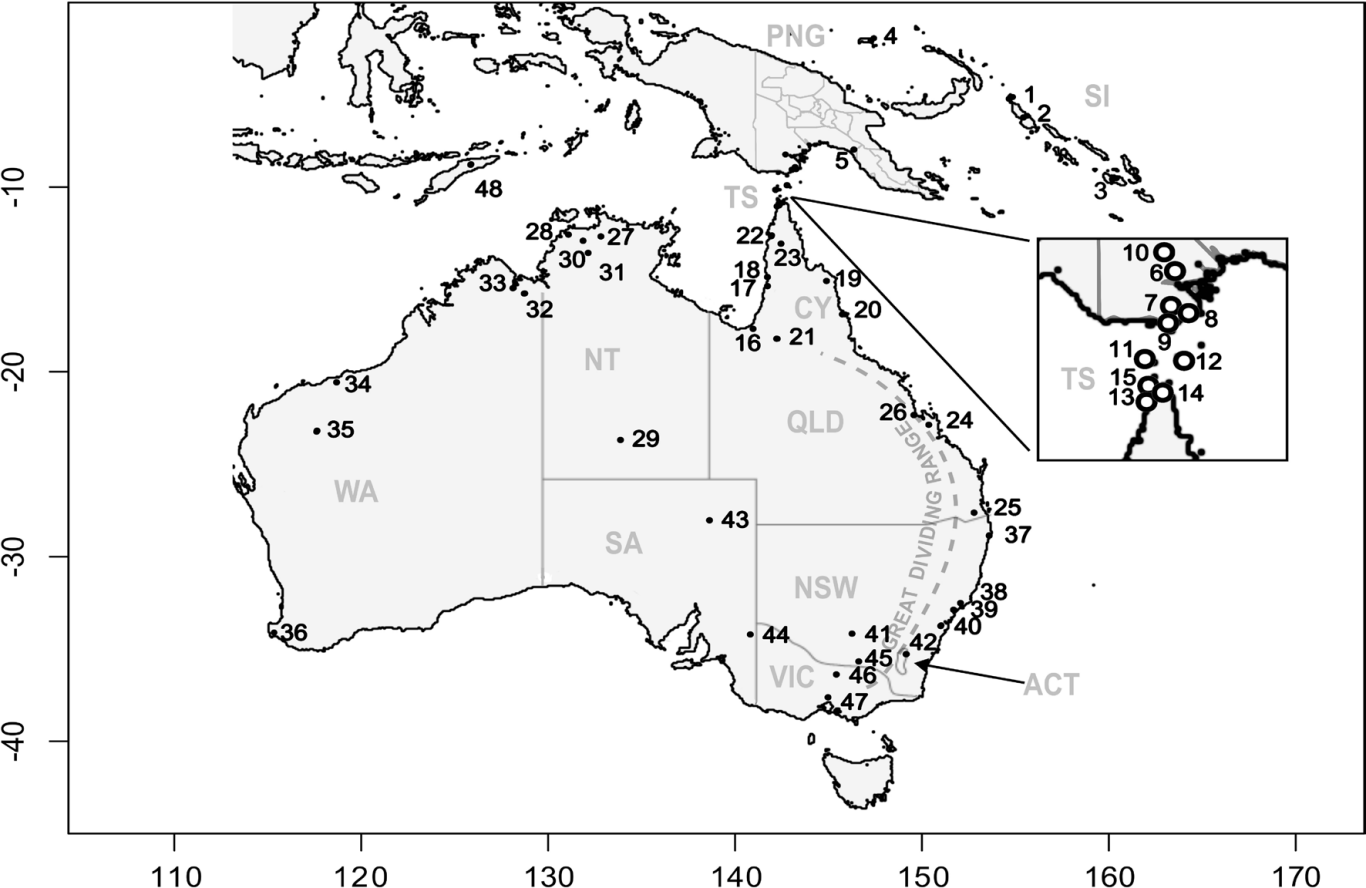
A line map of Australasia, displaying state boundaries and notable geographic features, with each point representing the sampling regions from which *Culex annulirostris* mosquitoes were collected. *SI* Solomon Islands, *QLD* Queensland, *WA* Western Australia, *NT* Northern Territory, *NSW* New South Wales, *SA* South Australia, *VIC* Victoria, *ACT* Australian Capital Territory, *TS* Torres Strait, *CY* Cape York. Map numbers refer to Table 1

*Culex annulirostris* belongs to the *Culex sitiens* subgroup which consists of three species distributed throughout the world, including *Culex palpalis* (Taylor) and *Culex sitiens* (Wiedemann), with the latter restricted coastally in Australia. Although members of the *Culex sitiens* subgroup can be difficult to distinguish using morphology alone [12, 22], a polymerase chain reaction (PCR) diagnostic can reliably separate these three species [9]. Mitochondrial DNA (mtDNA) sequencing of the *cytochrome oxidase* *1* (*COI*) has shown *Cx. annulirostris* to consist of multiple mtDNA lineages in Australia, Papua New Guinea (PNG) and the Solomon Islands (SI), with some lineages exhibiting additional geographic population structure [21]. Nuclear marker studies using allozymes and single locus DNA sequencing have also indicated that this mosquito may consist of reproductively isolated cryptic species in southern PNG and the Torres Strait Islands—a group of islands between southern PNG and north Queensland (see “TS” in Fig. 1) [13, 20]. In the Hemmerter et al. [20] study, a common lineage within “*Cx. annulirostris*” shows a wide distribution through PNG, Australia and the Solomon Islands. A second monophyletic mtDNA lineage, called *Cx. annulirostris* “PNG-1”, appeared geographically restricted to PNG, the Torres Strait Islands and the top of Queensland’s Cape York Peninsula region. Both lineages occur in areas of past JEV activity on the Torres Strait Islands and in PNG [25, 47, 48].

Historically, *Cx. annulirostris* has been implicated as the major vector for JEV in PNG and the Torres Strait Islands, with the first detection of JEV activity on these islands in 1995 [18]. Since then, intermittent JEV activity has been detected in the Torres Strait, with occasional appearances on the Australian mainland in the northern Cape York Peninsula in 2004 [46]. Additional evidence has emerged of more recent animal seroconversions in this region [6]. The distribution of historical JEV activity correlated well with the distribution of the PNG-1 lineage identified in Hemmerter et al. [21]. Because of this co-incidence, it was hypothesized that the PNG-1 lineage could be the primary vector of the virus. However in 2022, a widespread outbreak of JEV occurred through much of mainland Australia which was associated with a strong La Niña climate event that provided ideal conditions for abundant mosquito and waterbird populations critical to transmission cycles with the detection of JEV in mosquitoes, sentinel animals, livestock, wildlife and humans across five states [35, 50, 51]. This outbreak resulted in the declaration of a Communicable Disease Incident of National Significance (CDINS) [51]. From this outbreak it is now apparent that the other Australian lineages of *Cx. annulirostris* are capable vectors of JEV.

Notwithstanding the role of *Cx. annulirostris* in transmission of JEV, the mosquito has been demonstrated to effectively transmit MVEV [31] and KUNV [44]. Major outbreaks of MVEV have occurred in the 1950s and 1970s together with occasional activity, typically concomitant with flooding and above average rainfall associated with La Niña influenced climatic events [43]. Until recently, the most significant activity of MVEV in south-eastern Australia since 1974 was attributed to abundant populations of *Cx. annulirostris* [10]. Similarly, the mosquito has been identified as a competent vector of RRV [30] and contributor to outbreaks of disease caused by this virus [23].

Since *Cx. annulirostris* is the primary regional arbovirus vector, a deeper understanding of the genetic diversity and population genetics of this mosquito is essential to better study the biology and ecology of arbovirus transmission through this region. Thus, we aim to further investigate the distinct mtDNA lineages of *Cx. annulirostris*, using nuclear microsatellite markers to assess whether they show evidence of population structure at the nuclear level, and if they constitute distinct species. Our overarching hypothesis is that *Cx. annulirostris* sensu stricto is a complex of cryptic species in Australasia, and that analysis of nuclear microsatellite data will reveal evidence of reproductive isolation between mtDNA lineages when populations are in sympatry. Finally, if there is evidence of cryptic species, we aim to also develop a PCR molecular diagnostic to distinguish them.

## Methods

### Samples and collection

The *Cx. sitiens* subgroup samples used in this study were collected between 1999 and 2023 in PNG, the SI and all states of Australia. All samples were obtained using CO_2_ baited traps, run overnight between dusk and dawn (Fig. 1). Further details are shown in Table 1 and Fig. S1. All samples were initially morphologically identified as *Cx. sitiens* Subgroup members using regionally specific taxonomic keys and other resources [22]. After DNA extraction using a salt precipitation method [9], samples were distinguished from closely related species and confirmed as *Cx. annulirostris* by polymerase chain reaction restriction fragment length profiling (PCR–RFLP) of the ribosomal DNA (rDNA) ITS1(Internal Transcribed Spacer 1) following the procedure outlined in Beebe et al. [9].

**Table 1:** Location of individual *Culex annulirostris* sampled and analysed for *Cytochrome Oxidase I *(*COI*) (*N* = 301) and Microsatellite (MSAT) (*N* = 463) identity used in this study

Map no.	Region	Location	*N* (COI)	*N* (MSAT)	Longitude	Latitude
1	Solomon Islands	Buka, Solomon Archipelago	6	35	154.6707	−5.43128
2	Solomon Islands	Bougainville Island, Solomon Archipelago	1	0	155.3949	−6.22976
3	Solomon Islands	Guadalcanal, Solomon Archipelago	1	0	160.1443	−9.57989
4	Manus	Manus Island, Papua New Guinea	25	30	147.2578	−2.0627
5	Central Province	Site 6, Papua New Guinea (Central Province)	68	19	146.3431	−7.96781
5*	Central Province	Site 36, Papua New Guinea (Central Province)	1	4	146.281	−8.178
5*	Central Province	Site 40, Papua New Guinea (Central Province)	4	19	146.1903	−8.16924
6	Western Province	Abam, Papua New Guinea (Western Province)	7	0	143.2032	−8.92126
7	Western Province	Boze, Papua New Guinea (Western Province)	4	15	143.0397	−9.06176
8	Western Province	Dorogari, Papua New Guinea (Western Province)	2	0	143.2496	−9.02595
9	Western Province	Balimo, Papua New Guinea (Western Province)	9	14	147.196	−9.46707
10	CY_TSI	Badu Island, Torres Strait	7	14	142.1659	−10.1583
11	CY_TSI	Yam Island, QLD (Torres strait)	1	0	142.7726	−9.90027
12	CY_TSI	Injinoo, QLD (tip of Cape York)	2	0	142.2411	−11.0422
13	CY_TSI	Bamaga, QLD, Australia (tip of Cape York)	3	9	142.39	−10.8889
14	CY_TSI	Seisia, QLD (tip of Cape York)	1	0	142.3701	−10.8492
15	CY_TSI	Umagico, QLD (tip of Cape York)	1	0	142.35	−10.8893
16	Queensland	Normanton, QLD, Australia	4	17	141.0649	−17.687
17	Queensland	Kowanyama, QLD, Australia	5	5	141.7319	−15.3656
18	Queensland	Pormpuraaw, QLD, Australia	2	8	141.6165	−14.8993
19	Queensland	Laura, QLD, Australia	0	9	144.273	−15.4956
20	Queensland	Cairns, QLD, Australia	0	6	145.7663	−16.923
21	Queensland	Croydon, QLD, Australia	4	0	142.22	−18.2172
22	Queensland	Weipa, QLD, Australia	0	8	141.846	−12.649
23	Queensland	Merluna, QLD, Australia	0	3	142.4528	−13.0648
24	Queensland	Shoalwater Bay Training Area, QLD, Australia	4	0	150.1497	−22.2801
25	Queensland	Ipswich, QLD, Australia	1	0	152.76	−27.6152
26	Queensland	St. Lawrence, QLD, Australia	0	21	149.5432	−22.3497
27	Northern Territory	Jabiru, NT, Australia	3	0	132.8344	−12.6749
28	Northern Territory	Humpty-Doo, NT, Australia	22	27	131.3745	−12.6446
29	Northern Territory	Alice Springs, NT, Australia	0	11	133.8806	−23.7
30	Northern Territory	Mary River, NT, Australia	2	12	131.7208	−12.6767
31	Northern Territory	Mt Bundey, NT, Australia	1	0	131.6752	−12.9077
32	Northern WA	Kununurra, WA, Australia	3	5	128.7417	−15.7681
33	Northern WA	Wyndham, WA, Australia	8	12	128.1147	−15.5003
34	Northern WA	Port Hedland, WA, Australia	3	5	118.5753	−20.3116
35	Northern WA	Paraburdoo, WA, Australia	3	0	117.6777	−23.2012
36	Southern Aus	Swan Coast, WA, Australia	0	12	115.1587	−34.2308
37	Eastern NSW	Ballina, NSW, Australia	2	0	153.5646	−28.8615
38	Eastern NSW	Port Stephens, NSW, Australia	9	10	151.7411	−32.7623
39	Eastern NSW	Maryland, NSW, Australia	19	20	151.6626	−32.8778
40	Eastern NSW	Sydney (Baulkham Hills), NSW, Australia	2	0	150.9792	−33.7534
40*	Eastern NSW	Sydney (Picnic Point), NSW, Australia	20	20	151.0062	−33.9693
41	Southern Aus	Griffith, NSW, Australia	1	19	146.0509	−34.2885
42	Southern Aus	Canberra, ACT, Australia	0	8	149.1281	−35.2835
43	Southern Aus	Mungeranie, SA, Australia	7	10	138.6642	−28.0178
44	Southern Aus	Mundic Creek, SA, Australia	4	11	140.8035	−34.2124
45	Southern Aus	Wodonga, VIC, Australia	8	20	146.887	−36.115
46	Southern Aus	Shepparton, VIC, Australia	8	20	145.405	−36.382
47	Southern Aus	Melbourne, VIC, Australia	12	10	144.9633	−37.814
48		Timor Leste	1	0	125.8777	−8.6003

*ACT* Australian Capital Territory, *QLD* Queensland, *CY_TSI* Cape York/Torres Strait Islands, *WA* Western Australia, *NSW* New South Wales, *SA* South Australia, *VIC* Victoria

^*^Two distinct collection sites within site 40

### Mitochondrial sequencing and analysis

Mitochondrial sequence data were obtained by PCR amplification of part of the *COI* gene, as specified in Hemmerter et al. [21], followed by Sanger sequencing in both directions. Sanger sequencing was performed by Macrogen (Seoul, Korea) using the same forward and reverse primers as in Hemmerter et al. [21]. The sequence data were aligned with previously available data from Hemmerter et al. [21] using the “Geneious Alignment” multiple alignment tool in Geneious Prime (Version 2022.2.2), resulting in a final (385 bp long) *COI* alignment of 301 *Cx. annulirostris* individuals sampled from across Australia, PNG and the Solomon Archipelago. Mitochondrial (*COI*) sequence diversity (Haplotype diversity, Hd, and Pi, π), Tadjima’s *D* and differentiation (pairwise *F*_*ST*_) between populations was assessed using DnaSP version 6.12.03 [39]. The relationships of the resulting haplotype groups were then visualized by building a Median Joining Network in the program PopArt version 1.7 [7].

### Microsatellite development, amplification and analysis

A set of 12 novel microsatellite markers for *Cx. annulirostris* were initially developed from 454 sequence data using the methods described in Seah et al. [42]. Primers for the 12 microsatellites developed are shown in Table S1. Data were generated for these markers by PCR amplification of 520 individuals identified as *Cx. annulirostris* by molecular diagnostics [9]. Conditions and reagent volumes and enzymes used in PCRs are the same as described in Seah et al. [42], and dyes associated with each marker can be found in Table S1. Excess primers were removed from the PCR products by enzymatic degradation using ExoI (New England Biolabs, USA) and Antarctic Phosphatase (New England Biolabs, USA) enzymes and products were sent to Macrogen (Seoul, Korea) for capillary electrophoresis. Alleles for each microsatellite marker were scored manually using the program GeneMarker version 2.6.3. Markers showing a lack of variability will be removed from the final data set and individuals missing data for four or more markers were removed prior to analysis. It should be noted that although the mitochondrial and nuclear microsatellite datasets contain individuals sampled from the same sites only a subset of individuals have been genotyped for both set of markers,

To assess population structure from microsatellite data, we used the Bayesian clustering program, STRUCTURE v.2.3.4 [38]. Initially, the program was run using the no admixture model with location priors assigned by collection site for 20 iterations of K1–K8 (300,000 generation burn-in, 200,000 generation sample). The CLUMPAK webserver and STRUCTURE Harvester [16, 33] were then used to assess the most likely *K* value based on the Evanno method [17], as well as major and minor modes for each *K* value. Based on results of initial analysis which showed evidence for strong population structure between the PNG-1 and Solomon Island groups and other populations, we then ran STRUCTURE on a subset of data containing only individuals assigned to the other three groups (*n* = 382). For this run, the admixture model was used with location priors based on sampling location for K1 to K6 (300,000 generation burn-in, 200,000 generation sample). As above, STRUCTURE results were assessed using CLUMPAK and the Evanno method.

The populations identified by STRUCTURE were designated in reference to the *COI* lineages described by Hemmerter et al. [20, 21] as N-AUS (inclusive of PNG-2), S-AUS, PNG-1 and SI. We used the R package mmod to estimate Jost’s *D* (a statistic similar to *F*_*ST*_ but better suited to microsatellite data) [28]. To visualize relationships between populations based on Jost’s *D*, the R package phanghorn [41] was used to assemble neighbour-joining trees. A discriminant analysis of principal components (DAPC) was performed in the R package adegenet [26, 27], with populations assigned in line with STRUCTURE results. We used the xvalDAPC function to determine the optimal number of principal components (PCs) to retain in the DAPC (number of PCs retained = 20).

### Molecular diagnostic development for identifying species

A PCR diagnostic was developed atop the findings of Beebe et al. [9] and Hemmerter et al. [20], which demonstrated intraspecific variation in the *COI*, ITS1 and *ACE-2* genes of *Culex* mosquitoes. Ninety samples of *Cx annulirostris* from Central Province, PNG were PCR amplified using the ITS1 forward and reverse primers described by Beebe et al. [9]. These additional samples were Sanger sequenced for the ITS1 using the same primers set as the PCR and cleaned up using the Antarctic Phosphatase method [42] and sent to for sequencing to Macrogen (Seoul, Korea). The resulting ITS1 sequences were examined, aligned and edited using Geneious Prime (version 2022.2.2) alignment software. Oligonucleotide primers were designed to newly identified, lineage-specific mutations in the sequenced ITS1 sequence (Table S2). An internal control was also developed based on the ribosomal *18S* small subunit (*SSU*) region that would co-amplify in all *Culex* species. The lineage-specific sequences were amplified using multiplexed PCR reactions, containing a set of five primers at different concentrations (Table S3) and assessed using DNA extractions from samples of *Cx annulirostris* and PNG-1 previously identified by *COI* sequences. This included 45 samples of *Cx. sitiens* subgroup members from: Central Province, PNG (*n* = 15); Humpty-Doo, Northern Territory (*n* = 5); St. Lawrence, Queensland (*n* = 5); and Wodonga, Victoria (*n* = 10). The PCR reaction mix for amplifying the samples is outlined in Table S3. The thermal cycler protocol was 95 °C for 3 min, then 25 repeats of 95 °C for 1 min, 59 °C for 1 min, and 72 °C for 1 min, then followed by 72 °C for 5 min and a final 4 °C for 1 min. The amplified products were then run on a 3.5% agarose gel containing 1.5ul Midori Green dye (Nippon Genetics) per 50 ml of agarose solution at 80 V for 45 min with Hyperladder II (50 bp, Bioline Aus) used as size reference. Final products were visualized using a transilluminator.

## Results

### Mitochondrial diversity and population structure

**Fig. 2:**
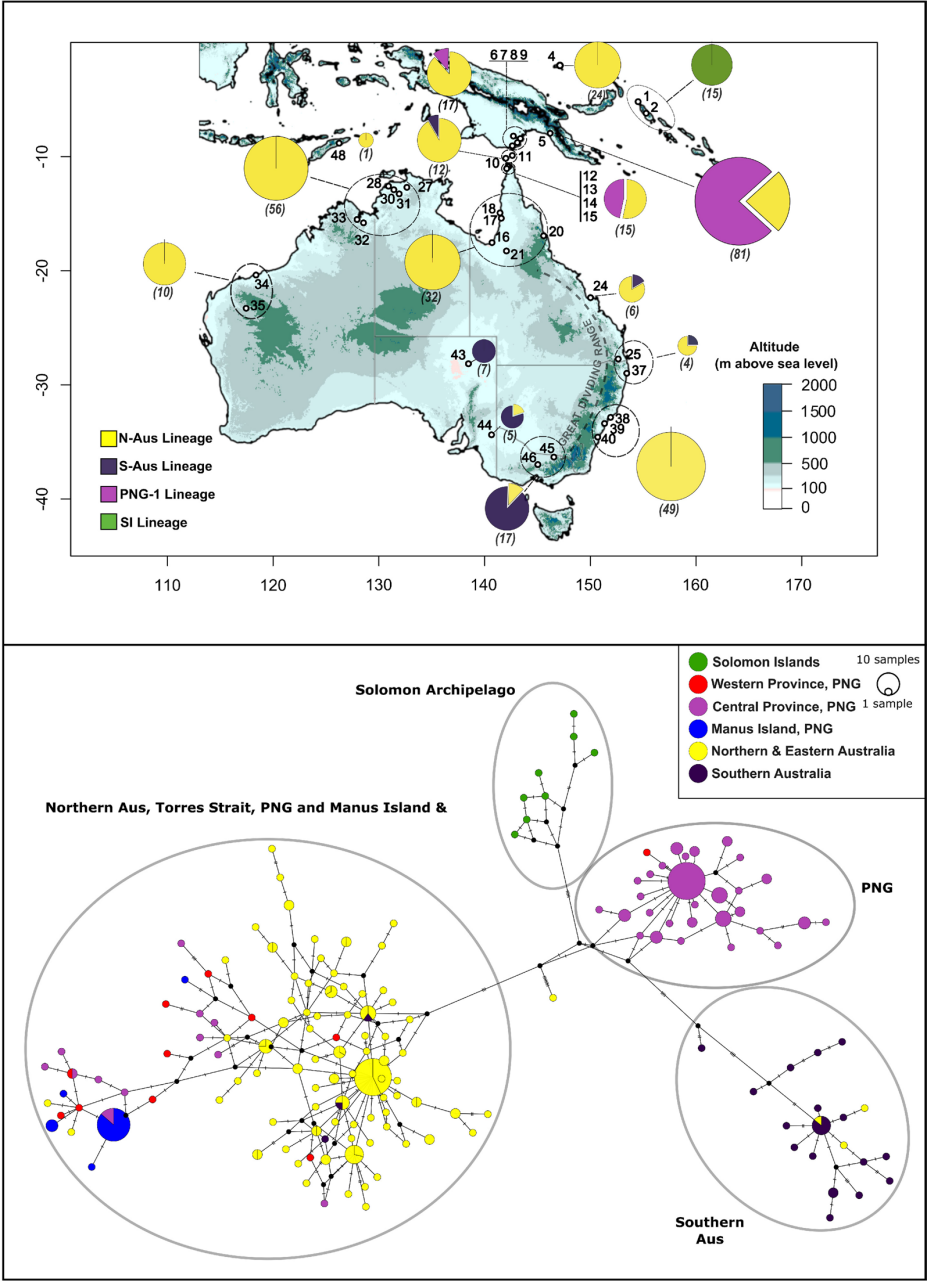
Top panel: A topographical map of Oceania showing the relative proportions of *Culex annulirostris* lineages sampled in different regions, based on *Cytochrome Oxidase* I (*COI*) mtDNA sequence data. Pie charts represent the percentage of individuals belonging to their respective *COI* lineage, from each sample area. Map numbers refer to Table 1. The number in brackets under each chart, as well as chart size represent the total number of sampled individuals from the area(s) indicated. Naming convention and mtDNA data adapted from ref. [21]. Bottom panel: A median-joining haplotype network of *Culex annulirostris* *COI *sequences. Each coloured circle represents a unique mitochondrial sequence (haplotype), with samples coloured by geographic region from which they were sampled. Circle sizes are proportionate to the number of individuals sampled for each haplotype, small black circles, dashes and lines represent unsampled haplotypes (polymorphisms)

The mitochondrial haplotype network shown in Fig. 2 shows the relative frequencies and relationships between the 301 *Cx. annulirostris* COI sequences sampled throughout the region. A total of 162 haplotypes were sampled overall (Hd = 0.98) and these sequences are available through GenBank (PP469228–PP469395). Samples were grouped and colour coded on the basis of their sampling location as follows: Queensland (QLD), Cape York and Torres Strait (CY_TSI), Northern Territory (NT), north Western Australia (NWA), eastern New South Wales (ENSW), South Australia, Victoria and western New South Wales (Southwest Aus), Western Province PNG (WPNG), Central Province PNG (CPNG), Solomon Islands (SI), Manus and PNG-1. See Table 2 for sample size and haplotype diversity information for each of these groups.

**Table 2:** Mitochondrial* COI* sequence diversity

Region	*N*	*N* haps	Hd	Pi (π$$)$$	Tajima’s *D*
Queensland	28		0.971	0.01218	−1.43
CY_TSI	14	13	0.989	0.01693	−1.60
Northern Territory	28	21	0.942	0.00838	−2.28**
Northern WA	17	13	0.949	0.00634	−1.57
Eastern NSW	52	33	0.943	0.00936	−1.23
Southern Aus	26	20	0.951	0.01568	−0.65
Central Province	32	6	0.389	0.01186	0.5
Western Province	19	17	0.982	0.01736	−1.31
Manus	25	5	0.420	0.00310	−1.81*
Solomon Islands	8	8	1.000	0.01011	0.05
PNG-1	51	30	0.971	0.00726	−1.82*
Total	301	162	–	–	–

*N* number of individuals sequenced, *N haps* number of haplotypes sampled, *Hd* Haplotype diversity

*<= 0.05; **<= 0.01

The haplotype network suggests that there are five major groupings present in the mitochondrial data corelating with (1) the northern Australian (N-AUS lineage) seen in the microsatellites which also includes samples from PNG, (2) the southern Australian (S-AUS) lineage, (3) the Solomon Islands (SI lineage), (4) the PNG-1 lineage and (5) a group from Manus Island made up of mostly the PNG-2 lineage as described in Hemmerter et al. [21]. Pairwise *F*_*ST*_ values support these groupings as shown in Table 3. Haplotype diversity was high in most groups, with a high proportion of singleton haplotypes sampled (Table 2). The N-AUS lineage sampled from the east coast of Australia, Northern Territory, Queensland and northern Western Australia also includes some samples from Manus Island, Western and Central Provinces, PNG, as well as the Torres Strait. This group forms the most complex part of the network, with the highest number of haplotypes sampled, as well as the most reticulation. However, it also contains the most individuals sampled meaning that this complexity and diversity could be an artefact of sample size. Pairwise *F*_*ST*_ was consistently low (< 0.05) between QLD, CY_TSI, NT, NWA and coastal NSW populations (Table 3), except for the pairwise comparison between Coastal NSW and NT (*F*_*ST*_ = 0.08), suggesting high connectivity between these regions. Within this group, there also appears to be some structure between PNG and Manus samples and the Australian samples, reflected in the relatively high *F*_*ST*_ values between these locations. There is almost complete lineage sorting between the two Australian groups, with the exceptions of three samples from southern Australia (one from Shepperton, Victoria, one from Wodonga, Victoria, and one from Mundic Creek, South Australia) possessing N-AUS haplotypes, as well as two samples from northern Australia (one from Ipswich, Queensland, and one from Shoal Water Bay, Queensland) and one sample from Torres Strait possessing a S-AUS haplotype. The southern Australian S-AUS population shows high *F*_*ST*_ values in all pairwise comparisons (> 0.5). The Solomon Islands group appears the most clearly distinct, shares no haplotypes with other groups and shows consistently high pairwise *F*_*ST*_ values in all comparisons (> 0.6). Finally, the PNG-1 group contains individuals sampled from Central province PNG, Western Province PNG with one individual sampled from the Cape York Peninsula in north Queensland. This PNG-1 group also has high *F*_*ST*_ values in all comparisons (> 0.5) apart from a moderate *F*_*ST*_ value in the pairwise comparison with Central Province, PNG (Table 4).

**Table 3:** Pairwise *F*_*ST*_ values between locations based on mitochondrial *COI* sequence data

	QLD	CY_TSI	NT	NWA	NSW	South_Aus	WPNG	CPNG	Manus	SI
QLD	0									
CY_TSI	0.00043	0								
NT	0.01623	0.03251	0							
NWA	0.02712	0.00748	0.0145	0						
NSW	0.0403	0.00432	0.08161	0.03112	0					
South_Aus	0.56528	0.54488	0.65004	0.67578	0.64214	0				
WPNG	0.17889	0.0581	0.2225	0.19643	0.19127	0.58094	0			
CPNG	0.46708	0.41624	0.53959	0.56073	0.52714	0.57906	0.34427	0		
Manus	0.62092	0.45901	0.68949	0.74316	0.65204	0.77961	0.35552	0.71081	0	
SI	0.6553	0.62441	0.71011	0.71905	0.70872	0.66837	0.64193	0.65915	0.85115	0
PNG_1	0.62421	0.5818	0.69716	0.72505	0.68469	0.64813	0.54259	0.12212	0.84843	0.7133

**Table 4:** Microsatellite diversity

Marker	Ho	He	Na	Len (bp)
CxAnn_TRI-3	0.475	0.792	17	176–221
CxAnn_TRI-4	0.406	0.600	10	182–209
CxAnn_TRI-5	0.524	0.677	11	174–204
CxAnn_TRI-8	0.625	0.827	13	165–195
CxAnn_TRI-9	0.491	0.702	10	171–198
CxAnn_TRI-11	0.661	0.703	11	137–179
CxAnn_TRI-14	0.500	0.747	13	123–165
CxAnn_DI-2	0.683	0.797	18	138–184
CxAnn_DI-8	0.065	0.107	8	132–150
CxAnn_TET-1	0.442	0.539	8	109–137

*Ho* observed heterozygosity, *He* expected heterozygosity, *Na* number of alleles, *Len* length range of amplified fragments

### Nuclear microsatellite population structure

**Fig. 3:**
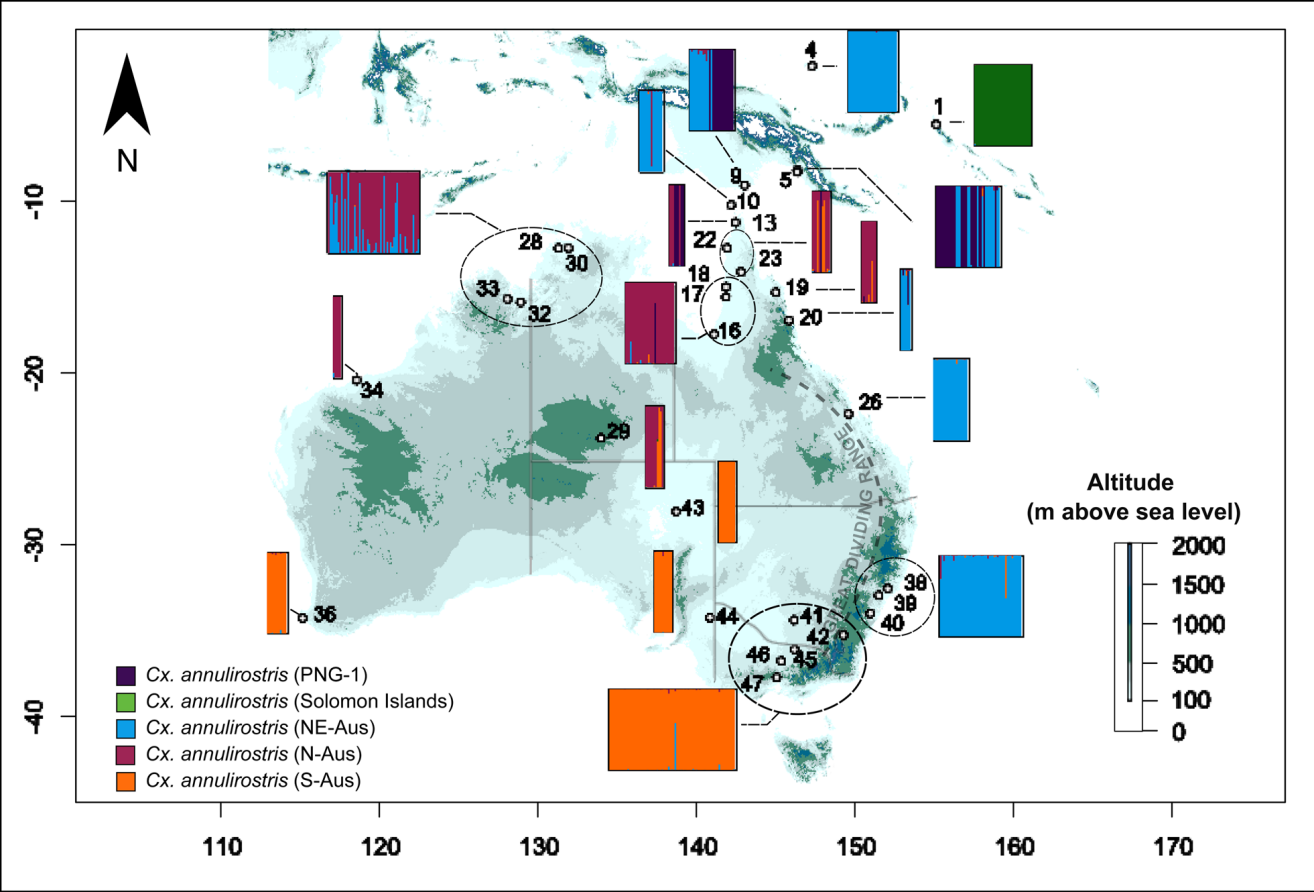
STRUCTURE plot generated from *Culex annulirostris* microsatellite data (10 loci) throughout Australasia. Results generated via STRUCTURE version (2.3.4), using the no admixture model *K* = 5, *N* = 463

Following removal of individuals missing data for four or more loci, the final microsatellite dataset contained 463 individuals. Two markers (TRI-1 and TRI-2) were removed due to lack of variation among samples, resulting in a final dataset of 463 individuals genotyped for ten markers. Results from the STRUCTURE analysis using the complete dataset and no admixture model suggested that *K* = 3 is the most likely level of population structure based on the Evanno method and based on the probability method is *K* = 8. The STRUCTURE plot for the major mode found for *K* = 5 from this analysis is shown in Fig. 3, revealing evidence of strong genetic structure between populations of *Cx. annulirostris* across the southwest Pacific region. The five genetic clusters identified were designated labels according to their relative mtDNA lineage distributions described by Hemmerter et al. [21], that being PNG-1, “Solomon Islands” (SI), northern Australia (N-AUS), and “Southern Australia” (S-AUS), with the addition of a cluster from northwestern Australia (NW-AUS). The dark purple cluster corresponds to the PNG-1 lineage (Fig. 3) and is mostly limited to mainland PNG, with a few individuals occurring at the tip of the Cape York Peninsula in northern Queensland. The Solomon Islands (SI) cluster (green) is found exclusively in the Solomon Archipelago. These two populations are clearly distinct from all other populations found across the Southwest Pacific, with all individuals belonging to these clusters showing a very high probability of assignment. The northern Australian (N-AUS) cluster (blue) is predominantly found in northern Australia (northern Western Australia, Northern Territory and Queensland), and in the south of Australia on the coastal side of the Great Dividing Range (Fig. 3). Additionally, some individuals from this population were sampled from PNG and the Torres Strait. Finally, the southern Australian S-AUS cluster (orange) occurs through southern Western Australia, South Australia as well as west of the Great Dividing Range in Victoria and New South Wales.

**Fig. 4:**
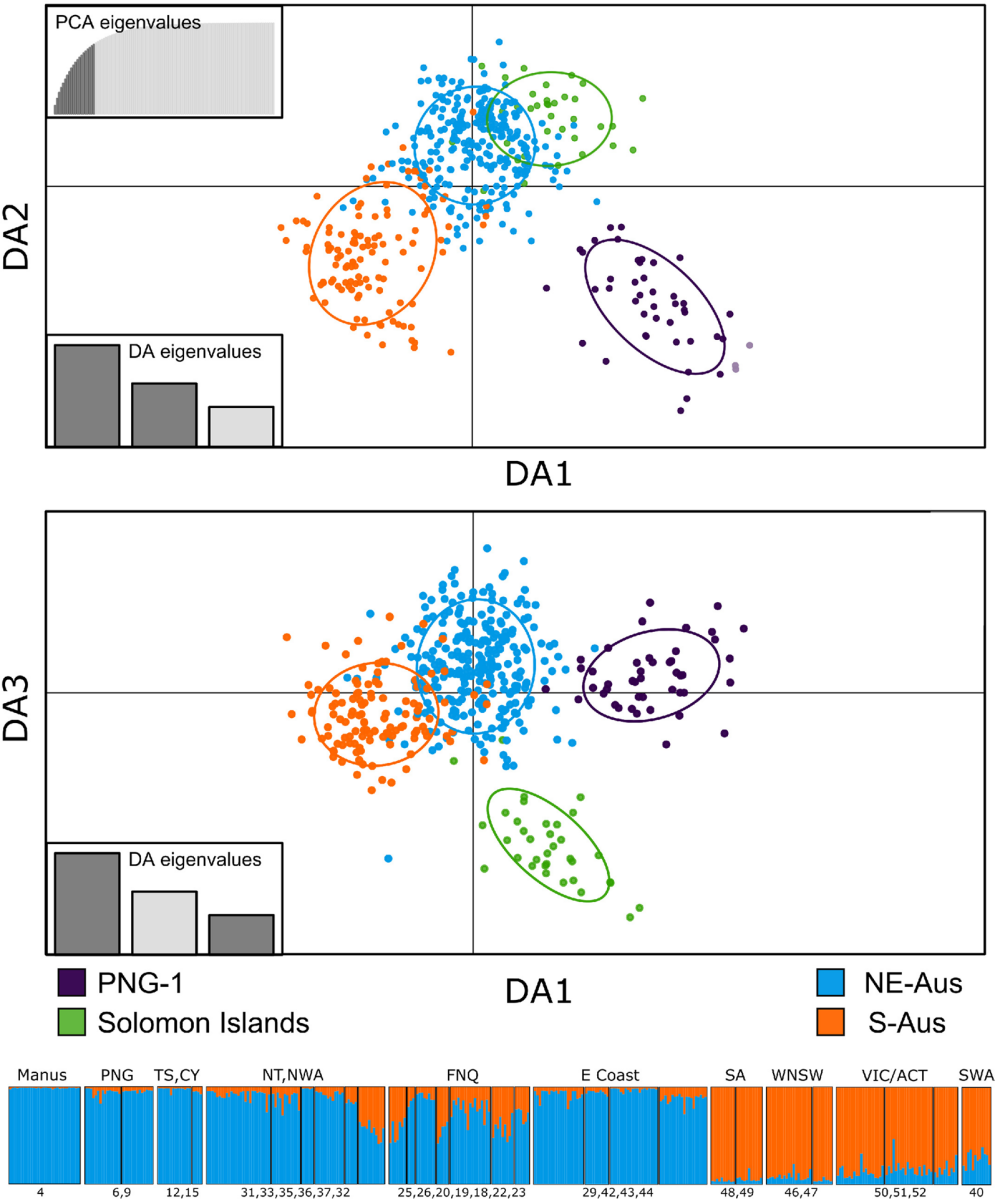
Population structure of *Culex annulirostris* based on nuclear microsatellite data (ten loci). Top panels: Discriminate analysis of principal components (DAPC) of *Culex annulirostris* *N* = 463, 10 loci and 20 retained principal components. Groups are coloured in accordance with STRUCTURE plots from Fig. 3 (PNG-1 and SI clusters) and the bottom panel of this Figure (NE-Aus and S-Aus). Bottom panel: STRUCTURE plot mapped to regions where samples were collected. Each bar is representative of one individual. The colours in each bar represent the proportion of ancestry of each individual to the populations assigned by the software. Map numbers shown below the plot refer to Table 1b. *Manus* Manus Island, *PNG* Papua New Guinea, *TS* Torres Strait Islands, *CY* Cape York Peninsula, *NT* Northern Territory, *NWA* northern Western Australia, *FND* far north Queensland, *E Coast* east coast of Australia, *SA* South Australia, *WNSW* western New South Wales, *VIC* Victoria, *ACT* Australian Capital Territory, *SWA* southern Western Australia

In addition to the no admixture model, STRUCTURE was also run using an admixture model on individuals belonging to the N-AUS, S-AUS and NW-AUS clusters (*N* = 384). The optimal *K* value found using the Evanno method was *K* = 2 and from the probability method was *K* = 5. Results from *K* = 2 are presented in Fig. 4 and while there is evidence for two largely distinct populations occurring in the north east and south of Australia, evidence of admixture is apparent between the N-AUS and S-AUS populations at site 32 in Alice Springs, central Australia as well as in far north Queensland (Fig. 4).

**Fig. 5:**
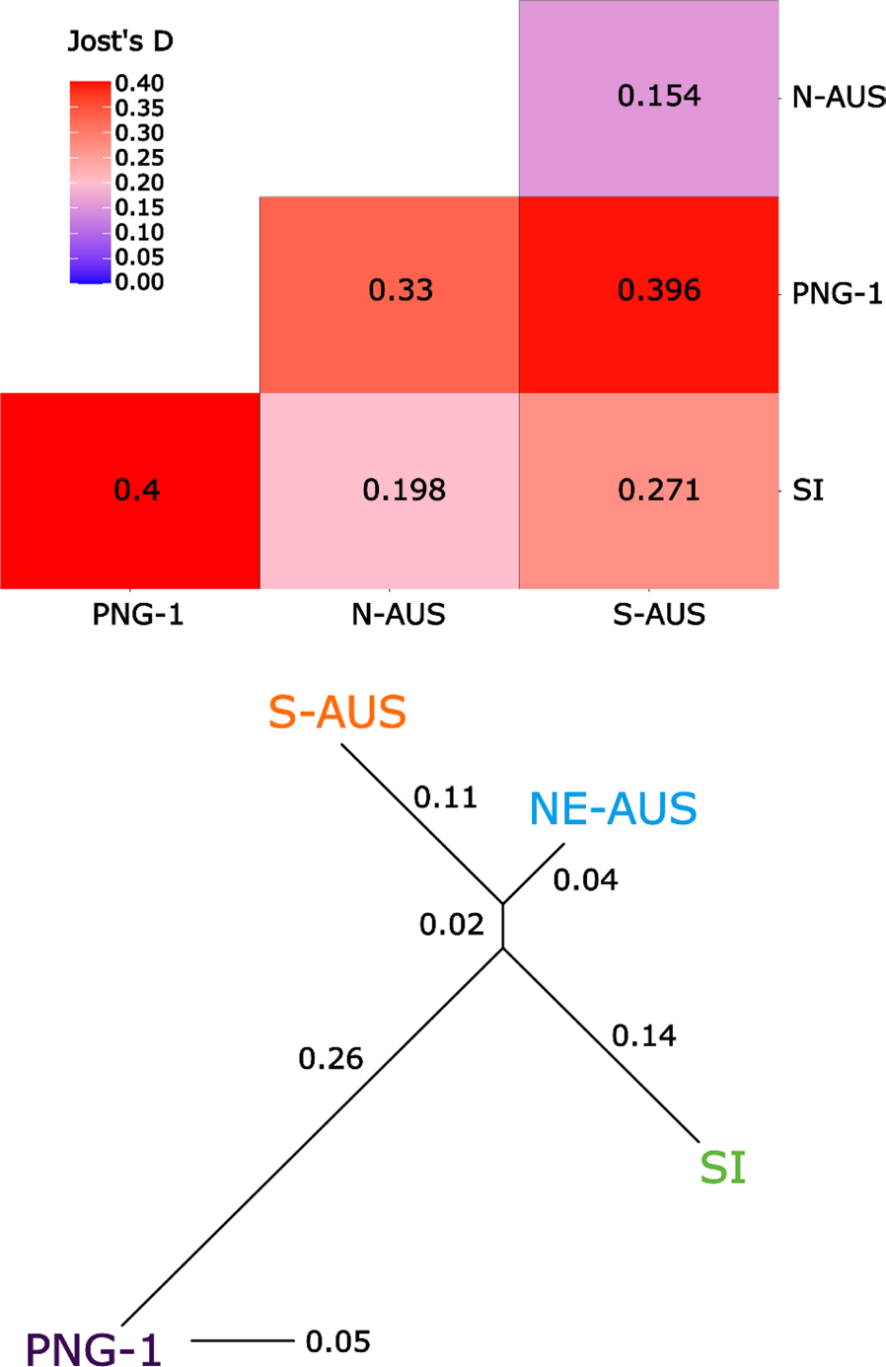
Top panel: Pair-wise matrix displaying Jost’s *D* [28] values generated from *Culex annulirostris* microsatellite data (ten loci). Values were estimated using R packages mmod 1.3.3 and adegenet 2.1.10. Bottom panel: Jost’s *D* matrix data represented as a neighbour joining tree

Results from DAPC largely agree with STRUCTURE analyses, and the four distinct population clusters identified from the STRUCTURE analysis differ in position across the three discriminant axes retained in the DAPC (Fig. 4). The northern Australia (N-AUS) population lies centrally on the two axes of greatest variance (DA1 and DA2), suggesting that it may be either the population from which others have dispersed and differentiated, or the most connected to the other populations. This agrees with estimates of Jost’s *D*, where the N-AUS population has the lowest average Jost’s *D* values in pairwise comparisons with other populations (Fig. 5). Both DAPC and pairwise Jost’s *D* support a close relationship between the S-AUS and N-AUS populations. Conversely, PNG-1 and Solomon Islands populations show clear differentiation from all other populations. The PNG-1 population is the most distinct based on both DAPC and Jost’s *D*. Although the Solomon Islands population is the most geographically isolated, it shows some degree of relatedness to the N-AUS and S-AUS clusters but is highly differentiated from PNG-1 (Fig. 5). Importantly, all individuals falling into the PNG-1 cluster for the STRUCTURE analyses that were also sequenced for the *COI* gene, belonged to the PNG-1 cluster in the mitochondrial haplotype network.

### Molecular diagnostic development for identifying species

**Fig. 6:**
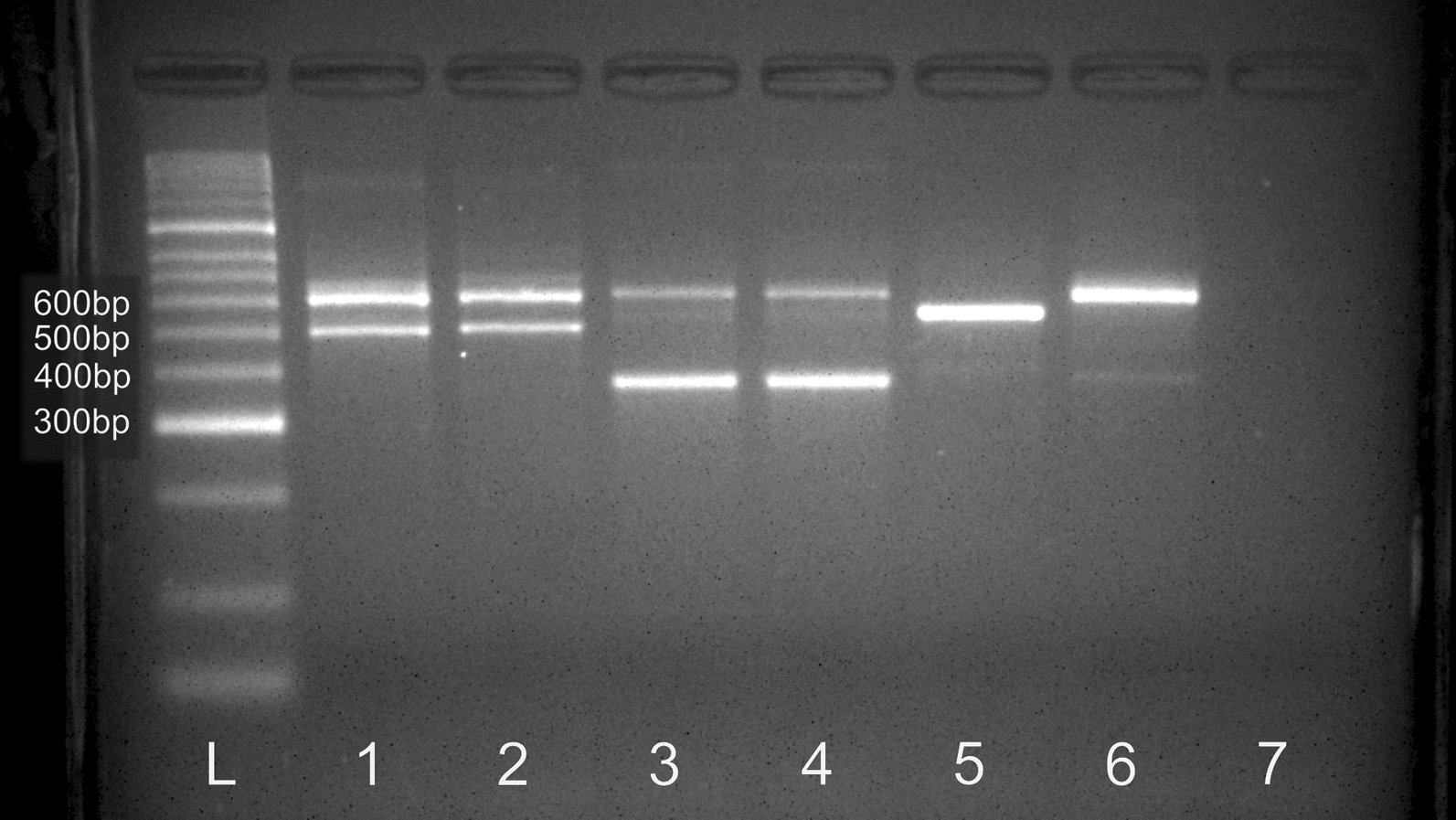
Agarose gel (3.5%) of the rDNA PCR diagnostic bands for *Culex sitiens* subgroup mosquito samples grouped by lineage. Lanes 1 and 2 are *Cx. annulirostris* North Territory and Queensland in Australia. Lanes 3 and 4 are *Cx. annulirostris* (PNG-1) from Central Province, Papua New Guinea. Lanes 5 and 6 are *Culex sitiens* and *Culex palpalis* showing only the positive control confirming the presence of gDNA, respectively. Lane 7 is a negative control with no DNA template

The utility and reliability of the allele-specific PCR diagnostic developed around sequence polymorphism in the ITS1 consensus sequence (GenBank accession number PP464078) was assessed using newly processed samples which had been identified prior to species by *COI *DNA sequence as *Cx. annulirostris*, *Cx. annulirostris* (PNG-1), *Cx. sitiens* or *Cx. palpalis*. The diagnostic reliably separated the PNG-1 lineage from *Cx. annulirostris* and did not amplify *Cx. sitiens* or *Cx. palpalis* DNA samples (Fig. 6). The PNG-1 samples produced amplicons approximately 400 bp and all other *Cx. annulirostris* samples (N-AUS, S-AUS, SI and PNG-2 mtDNA lineages) produced products closer to 500 bp in length. The *18S* internal control amplified products in all samples regardless of species/lineage. An *18S* amplicon product size difference was found between the *Cx. annulirostris* and *Cx. palpalis* samples (650 bp) and the *Cx. sitiens* samples generating slightly smaller band that permitted *Cx. sitiens* to be distinguished by the internal control. In the assessment of 45 field samples collected from PNG, previously identified by COI sequence, 100% showed agreement with their ability to distinguish *Cx. annulirostris* from the PNG-1 lineage.

## Discussion

In this study we developed 12 microsatellite markers for *Cx. annulirostris*, of which 10 showed sufficient variation to be of utility for population genetics analysis. For these 10 markers, 463 *Cx. annulirostris* samples from 53 sites across Australasia were genotyped and analysed revealing clear population structure both within and between landmasses in Australasia. We found four major population clusters that correlate with previously identified mtDNA lineages. Two of these populations occur within mainland Australia (i.e. N-AUS and S-AUS) with possible admixture evident between these clusters in some regions, including central Australia and far north Queensland, indicating that reproductive isolation between these populations may not be complete. The Northern Australian N-AUS population extends throughout northern and eastern Australia and into New Guinea. This population appears genetically isolated from the PNG-1 mtDNA lineage even when in sympatry, suggesting reproductive isolation and cryptic species status of the PNG-1 lineage.

A species diagnostic PCR was developed to lineage-specific sequence variation present in the fast-evolving and high genome copy number nuclear rDNA ITS1. The diagnostic includes a conserved rDNA *18S* internal control allele that consistently amplifies all *Cx. sitiens* subgroup members. This diagnostic clearly distinguishes PNG-1 from *Cx. annulirostris* with no cross-reactivity observed from *Cx palpalis* or *Cx. sitiens*. Coincidentally, *Cx. sitiens* can also be distinguished from the other *Cx. sitiens* subgroup members by its smaller *18S* PCR product observed in the internal control. The genetically distinct SI population in the Solomon Islands is also identified as *Cx. annulirostris* s.l. by the PCR diagnostic. It is likely that this population is highly differentiated because of its geographic isolation, and while this population may represent a distinct undescribed species, its geographical isolation makes it difficult to determine whether this is the case.

### Population structure and relationships of *Cx. annulirostris* in the Southwest Pacific

The *COI* haplotype network presented in Fig. 2 provides insights into the relationships between mitochondrial lineages of *Cx. annulirostris* within Australasia. The high haplotype diversity (Hd > 0.95) in the Australian lineages indicate that these population have likely been present and relatively stable for a long time. Whilst mtDNA is useful for making broad inferences about the population structure of organisms, its maternal inheritance means that in order to demonstrate reproductive isolation, it is important to supplement mitochondrial data with other lines of evidence, such as nuclear markers that undergo meiotic recombination. Previous findings by Hemmerter et al. [21] found distinct monophyletic and co-occurring mtDNA lineages (the Australian lineage and PNG-1 lineage) that were also supported by the presence of nuclear *acetylcholinesterase 2* gene sequences lineages for *Cx. annulirostris* and the PNG-1 [20]. Authors hypothesized that these lineages were likely reproductively isolated and constitute separate cryptic species. Our study followed up on this hypothesis with nuclear microsatellite markers and confirmed reproductive isolation between *Cx. annulirostris* and the PNG-1.

The nuclear markers used in this study separate *Cx. annulirostris* s.l. into four main genetic clusters, mostly in accordance with previous mtDNA sequence-based findings [20, 21]. However, unlike the mtDNA, there is evidence of admixture between the northern and southern Australian clusters, particularly in central Australia and far north Queensland, possibly suggesting gene flow and reproductive compatibility when these populations come into contact as seen by admixture in some individual from central and north-east Australia (Fig. 3). These two populations may represent nascent species with ongoing hybridization in areas where they overlap, as was observed in the *Cx. pipiens* complex [1, 19], and thus could remain independently evolving metapopulations that could be regarded as species [15]. Both the DAPC and Jost’s *D* performed on the northern and southern Australia clusters exhibit high degrees of similarity and low levels of differentiation, respectively (Jost’s *D* = 0.11), showing that relative to other populations, they are each other’s closest relatives. Populations in Sydney and Newcastle, NSW, are relatively far south in Australia yet they appear to be genetically connected with high levels of gene flow to those found across northern Australia, observed in both nuclear microsatellites and mtDNA (Figs. 2, 3). This extensive genetic connectivity across northern Australia and down the east coast is, despite nearby populations on the other side of the Great Dividing Range (sites 46–52), being clearly distinct and belonging to the southern Australian lineage although occuring at comparable southern latitudes such as Shepparton and Wodonga, Victoria.

It is likely that a few processes are driving the population structure within Australia. Firstly, a north–south latitudinal climate divide exists through Australia where the northern Australia open savanna region encounters a strong yearly tropical monsoonal influence where 90% of the rain occurs the wet season (December–April), while rainless periods in the dry season can be measured in weeks and temperatures and humidity are lower [8]. These yearly population crashes and expansions may drive differences in allele frequencies in the affected population over time [37], although the latitudinal differences in the climate between northern Australia and southern Australia may also be contributing to population structure. The Great Dividing Range appears also to be acting as a physical barrier to dispersal, separating the south-east coastal and inland populations. This extensive mountain range may represent an ecological as well as physical barrier, as there is a general absence of extensive freshwater wetlands across this region, and temperatures are generally lower during summer months than either coastal or inland regions. Additionally, climatic conditions on either side of the range are different, especially in terms of rainfall and temperature fluctuations. The coastal region to the east of the Great Dividing Range experiences higher and more consistent rainfall within and between years, and less seasonal fluctuation in temperature in the areas to the west of the range [24]. Thus, populations on either side of the range may be adapted to the different climates in these locations and the abundance of *Cx. annulirostris* is influenced by thermal conditions, but most importantly rainfall [29]. Thus, the differing temperatures and rainfall on either side of the Great Dividing Range may have driven distinct adaptive traits like thermal tolerance or desiccation resistance.

Despite *Cx. annulirostris* being documented dispersing widely, potentially more than 12 km, from larval habitats [11], and up to 200 km when wind dispersed [32], mountain ranges present barriers to dispersal to many species, frequently resulting in population differentiation. Similar findings to this were identified in previous studies of *Aedes aegypti* in Mexico, Central America and Peru, South America, in which *Ae. aegypti* mtDNA haplotypes were found to differ on either side of notable regions of high elevation [14]. Several *Anopheles* mosquitoes in New Guinea also show evidence of strong population structure likely evolving as a result of dispersal barriers presented by elevational barriers [2–5].

Viewing the genetic relationships between the genetic clusters identified in this study, the northern and southern Australian populations appear central to the other two clusters, with PNG-1 the most distant. The central location and the high diversity observed in the Australian populations may suggest that these represent the ancestral populations from which other groups have differentiated. The notion that PNG-1 is genetically different from other *Cx. annulirostris* groups is consistent with the findings from Hemmerter [20] using the nuclear *ace2 *gene and the earlier observations of Chapman et al. [12, 13]. Indeed the combined evidence from nuclear and mtDNA markers suggest a highly differentiated lineage of mosquitoes, and the apparent lack of admixture when in sympatry with *Cx. annulirostris* provides strong evidence that the PNG-1 lineage is a separate species. The Solomon Islands (SI) cluster appears most closely related to the AUS populations but is geographically isolated and may also represent a distinct species as well. The S-AUS cluster is genetically more related to the N-AUS population and some potential admixture is observed, especially in central Australia and far north Queensland. Clarification of the species status of the two Australian genetic clusters will likely require more sampling and analyses of inland populations in western Queensland and central Australia, as well as additional genomic and morphological data.

### Implications of a cryptic *Culex* species in the Southwest Pacific

The finding that the PNG-1 lineage is a separate species to *Cx. annulirostris* is significant as it will likely show a different ecology, biology, behaviour and vectorial capacity. The PNG-1 lineage appears more common in southern PNG than *Cx. annulirostris* and currently shows a southern limit coinciding with historical JEV detection [21]. This PNG-1 lineage has probably been involved in the flavivirus activity detected in the Western Province of southern PNG [25] and is likely capable of transmitting the same medically important arboviruses as *Cx. annulirostris*. The correlation between the range of the PNG-1 lineage and historical JEV activity led to the hypothesis that it may be responsible for cycling the virus to the exclusion of Australian *Cx. annulirostris* [21, 45]. This hypothesis may have been influenced by the finding that Australian that *Cx. annulirostris* prefers blood-feeding on marsupials, potentially diverting virus transmission away from important amplifying hosts like wading birds and pigs [49]. Further investigations into the host preferences of the different lineages are necessary to better understand the recent JEV outbreak throughout Australia in 2022. With a warming climate we expect to see a southern expansion of the PNG-1 species further into northern Australia, joining the other recent invasive exotic arbovirus vectors *Cx. gelidus* Theobald [36] and *Cx. tritaeniorhynchus* Giles [34] in establishing on mainland Australia [50].

## Conclusions

This study adds to the growing body of work suggesting that the taxon presently known as *Cx. annulirostris*, consists of at least two cryptic species. The PNG-1 lineage and N-AUS lineages have been observed reproductively isolated using allozymes [13], nuclear DNA sequencing [20], the microsatellites presented here as well as the rDNA ITS1-based diagnostic. These putative species co-occur in northern Australia and New Guinea, lending further weight to their likely species status. The Solomon Islands population may also represent a distinct species, but its geographic isolation makes it hard to assess whether it would be reproductively isolated from other population. Additionally, we find further nuclear evidence of population structure between geographic regions in Australia and New Guinea for the AUS lineage which may hint at additional subspecies. It should be noted that the nuclear data used in this study were based on ten microsatellite markers which do have limitations, including the potential for homoplasy. We expect that future work on the species (complex) using whole genome sequencing or RADseq will reveal similar but stronger genetic structure and relationships as what we have observed in this study.

## Supplementary Information

Supplementary material 1: Table S1. Microsatellite primer sequences.

Supplementary material 2: Table S2. Species specific ITS1 and internal control primer sequences.

Supplementary material 3: Table S3. Species specific PCR diagnostic reaction mix (per sample).

## Data Availability

Data availability The Culex annulirostris PNG-1 ITS1 lineage consensus sequence can be obtained from GenBank accession number PP464078. Additional COI sequences can be obtained from GenBank accession number PP469228–PP469395.
