# Supplementary material for: Nuclear and mitochondrial population genetics of the Australasian arbovirus vector Culex annulirostris (Skuse) reveals strong geographic structure and cryptic species
Source: Parasit Vectors. 2024 Dec 4;17:501. doi: 10.1186/s13071-024-06551-8 (PMC11619117; doi:10.1186/s13071-024-06551-8)
Supplement: Supplementary file 1 — Supplementary material 1: Table S1. Microsatellite primer sequences. [file 13071_2024_6551_MOESM1_ESM.docx]

**Table S1:** Microsatellite primer sequences.

| **Primer Set** | **Forward Sequence** | **Reverse Sequence** |
| --- | --- | --- |
| CxAnn_TRI-1 | 5’-GTAAAACGACGGCCAGTTTCTGAACGCTGTGAAGCC-3’ | 5’-TTCTTCAGCTCGACCTCCAC-3’ |
| CxAnn_TRI-2 | 5’-GTAAAACGACGGCCAGTTCTAGACCATCGACGGCAG-3’ | 5’-TTAGGCCTACAGAAGCACCG-3’ |
| CxAnn_TRI-3 | 5’-GTAAAACGACGGCCAGCTGTTGCAGTTGAGTCCTGG-3’ | 5’-CCCGTTCAATTGGCTACTGATTG-3’ |
| CxAnn_TRI-4 | 5’-GTAAAACGACGGCCAGCGCCATTGATGACGTGCTTC-3’ | 5’-CCTCGCTCTTCTTCTTACCACC-3’ |
| CxAnn_TRI-5 | 5’-GTAAAACGACGGCCAGCTCAGCAAGGCCAATCGAAG-3’ | 5’-TTGAACGGTAGCAGCGTTTC-3’ |
| CxAnn_TRI-8 | 5’-GTAAAACGACGGCCAGCCTTCAATCAGGCCCAATCTG-3’ | 5’-GATGCCTGCACCGGAAAG-3’ |
| CxAnn_TRI-9 | 5’-GTAAAACGACGGCCAGTATACAAACGAACGGCAGCG-3’ | 5’-TCCCGAGACTGTGAGGTTATG-3’ |
| CxAnn_TRI-11 | 5’-GTAAAACGACGGCCAGGCAGAACCCGCTCATACATC-3’ | 5’-CACACATGAGAAATCCACGGG-3’ |
| CxAnn_TRI-14 | 5’-GTAAAACGACGGCCAGTATCTCGGCCAGTGGTAAG-3’ | 5’-CTCTCTCCCACGGATGATGG-3’ |
| CxAnn_DI-2 | 5’-GTAAAACGACGGCCAGCACATGTCGAGAAGCTGAGC-3’ | 5’-AGGAACGTGACATCCTTGGG-3’ |
| CxAnn_DI-8 | 5’-GTAAAACGACGGCCAGGAACCGGACAAATCTCCCAC-3’ | 5’-ATTTCGCTTGCTGTCTGGTC-3’ |
| CxAnn_TET-1 | 5’-GTAAAACGACGGCCAGCGAAGTCACCACCAACACTG-3’ | 5’-ATGTGTCCTGCAGTTCACACA-3’ |
