# Supplementary material for: Nuclear and mitochondrial population genetics of the Australasian arbovirus vector Culex annulirostris (Skuse) reveals strong geographic structure and cryptic species
Source: Parasit Vectors. 2024 Dec 4;17:501. doi: 10.1186/s13071-024-06551-8 (PMC11619117; doi:10.1186/s13071-024-06551-8)
Supplement: Supplementary file 2 — Supplementary material 2: Table S2. Species specific ITS1 and internal control primer sequences. [file 13071_2024_6551_MOESM2_ESM.docx]

**Table S2.** Species specific ITS1 and internal control primer sequences.

| Primer Name | Sequence | Fragment Size (bp) |
| --- | --- | --- |
| Cx-ITS1-39f | 5’- TTGTACACACCGCCCGTCGC - 3’ | - |
| Cx-PNG-428r | 5’- CTCCCTAGGCTGGTCAGGTC - 3’ | ~400bp |
| Cx-Ann-570r | 5’- CAGTAAAAACCCCAAAAC - 3’ | ~550bp |
| SSU-3870f | 5’- GGATTATTTAGTGAGGTCTTTGAAGG - 3’ | - |
| SSU-4499r | 5’- GCAGTTCACATTCTGACGCG - 3’ | ~650bp |
