# Supplementary material for: Nuclear and mitochondrial population genetics of the Australasian arbovirus vector Culex annulirostris (Skuse) reveals strong geographic structure and cryptic species
Source: Parasit Vectors. 2024 Dec 4;17:501. doi: 10.1186/s13071-024-06551-8 (PMC11619117; doi:10.1186/s13071-024-06551-8)
Supplement: Supplementary file 3 — Supplementary material 3: Table S3. Species specific PCR diagnostic reaction mix (per sample). [file 13071_2024_6551_MOESM3_ESM.docx]

**Table S3**: Species specific PCR diagnostic reaction mix (per sample).

| Reagent | Concentration | Amount |
| --- | --- | --- |
| MyTaq Reaction Buffer | 5x | 4uL |
| Ultra-Pure H_2_O | - | 16uL |
| Cx-ITS1-39f | 1x (100pmol) | 0.1uL |
| Cx-PNG-428r | 1x | 0.1uL |
| Cx-PNG-570r | 1x | 0.1uL |
| SSU-3870f | 1x (100pmol) | 0.05uL |
| SSU-4499r | 1x | 0.05uL |
| MyTaq Polymerase | 10U/µL | 1U |
| Extracted gDNA | 5-35ng/µL | 1uL |
| Total Volume |  | ~21uL |
